# RCSB PDB *Mobile*: iOS and Android mobile apps to provide data access and visualization to the RCSB Protein Data Bank

**DOI:** 10.1093/bioinformatics/btu596

**Published:** 2014-09-02

**Authors:** Gregory B. Quinn, Chunxiao Bi, Cole H. Christie, Kyle Pang, Andreas Prlić, Takanori Nakane, Christine Zardecki, Maria Voigt, Helen M. Berman, Philip E. Bourne, Peter W. Rose

**Affiliations:** ^1^RCSB Protein Data Bank, San Diego Supercomputer Center, University of California San Diego, La Jolla, CA 92093-0743, USA, ^2^Graduate School of Medicine, Kyoto University, Yoshidakonoecho, Sakyo Ward, Kyoto, Kyoto Prefecture 606-8501, Japan, ^3^RCSB Protein Data Bank, Center for Integrative Proteomics Research, Rutgers, The State University of New Jersey, Piscataway, NJ 08854-8087 and ^4^Skaggs School of Pharmacy and Pharmaceutical Sciences, University of California San Diego, La Jolla, CA 92093-0743

## Abstract

**Summary:** The Research Collaboratory for Structural Bioinformatics Protein Data Bank (RCSB PDB) resource provides tools for query, analysis and visualization of the 3D structures in the PDB archive. As the mobile Web is starting to surpass desktop and laptop usage, scientists and educators are beginning to integrate mobile devices into their research and teaching. In response, we have developed the RCSB PDB *Mobile* app for the iOS and Android mobile platforms to enable fast and convenient access to RCSB PDB data and services. Using the app, users from the general public to expert researchers can quickly search and visualize biomolecules, and add personal annotations via the RCSB PDB’s integrated MyPDB service.

**Availability and implementation:** RCSB PDB *Mobile* is freely available from the Apple App Store and Google Play (http://www.rcsb.org).

**Contact:** pwrose@ucsd.edu

## 1 INTRODUCTION

The popularity of mobile computing devices such as smart phones and tablets ensures that they play significant roles in our daily lives. The CPU speed, memory resources and graphics display capabilities of these devices have become increasingly powerful and sophisticated and can support advanced applications whether at work, home or on the go. An early example of such an app is the Hematopoietic Expression Viewer (James *et al.*, 2012), which provides access to microarray gene expression data.

Access to data- and content-rich life science resources such as the Protein Data Bank (PDB) (Berman *et al.*, 2003) through the Research Collaboratory for Structural Bioinformatics (RCSB) PDB Web site (Berman *et al.*, 2000, Rose *et al.*, 2011, 2013) can, however, prove problematic on tablets, and more so on hand-held devices, such as smart phones. Even with adaptively written Web sites that auto-format content for the mobile device screen dimensions, Web site content can be difficult to navigate using pinch-to-zoom capability of mobile platforms. As a result, customized apps have become popular, using mobile operating system's platform native widgets and capabilities to provide responsive and easily navigable information pathways.

Another mobile-specific challenge involves biomolecular structure visualization on mobile platforms such as iOS and Android. A comprehensive feature comparison of available viewers is available (Yiu and Chen, 2014). These platforms do not support Java Applets such as Jmol (Hanson, 2010), and their CPU capability is often insufficient to achieve acceptable responsive interactivity with software-based rendering. One possible work-around would be the use of WebGL, a mechanism that provides the ability to use hardware-accelerated 3D rendering through a Web browser; however, it is not fully supported on all mobile platform browsers.

Following requests from our users, who have iOS and Android devices, we initiated the development of native clients.

## 2 METHODS AND FEATURES

Our goal was to produce an intuitive app with the following core capabilities: (i) simple search interface, (ii) quick browsing of search results, (iii) view of basic data about a structure entry and its PubMed abstract, (iv) high-performance molecular visualization and (v) access to the archive of *Molecule of the Month* educational articles by David Goodsell (Dutta *et al.*, 2010).

Analysis of RCSB PDB Web site statistics when we initiated development efforts showed that iOS access far outstripped Android site visits. Moreover, our experience with development tools and API suggested that iOS would to be a significantly more robust system, with fewer device form factors to support and less operating system fragmentation. Therefore, iOS was chosen as the first development project, followed by development of the native Android app.

Development environments for the iOS version of the app used Xcode 5 and version iOS 7. Development of the Android app version used Eclipse and the Google Android Development Kit (ADK) Eclipse plug-in, together with the Java SDK.

### 2.1 Workflow

**Fig. 1.**
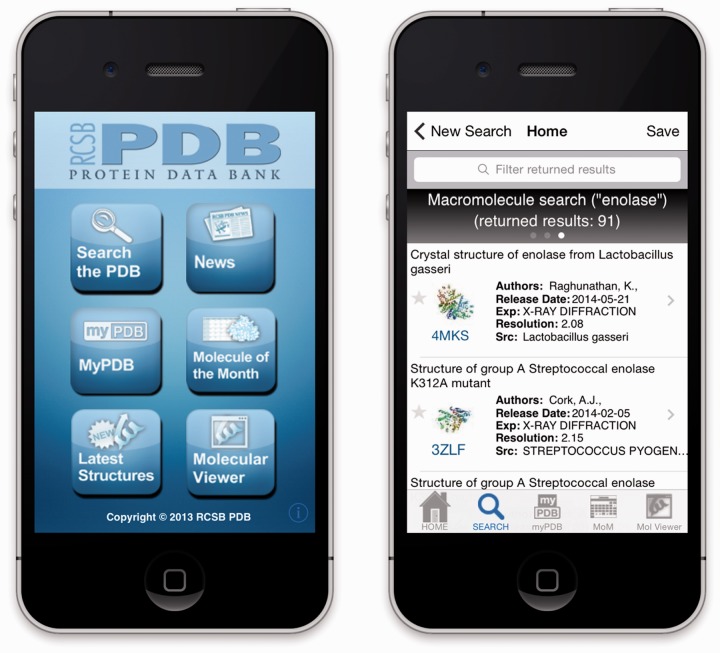
Home screen and search results listing on an iPhone

The visual design and workflow of the application are the same for both the iOS and the Android versions. The home screen (Fig. 1) provides the entry point to six options for biomacromolecular exploration.

### 2.2 Data access

Data access classes use the published RESTful Web services (Rose *et al.*, 2011), with the exception of the weekly update of the internal index and MyPDB interactions.

A key feature of the app, the ability to quickly display search data with minimal network bandwidth overhead, was achieved in part with a minimal SQLlite-formatted internal index of PDB data on the mobile device. With this approach, queries to our RESTful Web services need only return a list of PDB IDs, thereby enhancing speed and reducing bandwidth requirements, an important attribute when using the app on a cellular connection. To support this functionality, the index is synchronized weekly with new data releases.

The PubMed Abstract screen is displayed after selecting an entry in a results listing. It also provides a pathway to viewing the entry in the included molecular viewer. Encapsulating data within the thumbnail images used in results listings mitigate the impact of multiple data requests to the server and resultant network latency. When the user selects an entry in a results listing, PDB entry metadata is extracted from the ‘Description’ EXIF field of the JPEG-formatted thumbnail image. That textual content is then injected by the application into the Web page template via Java-Script. The internal index provides basic information and the first author of a structure entry (Fig. 1). Data included in the thumbnail comprise the PubMed abstract (where available), the full author list and other entry metadata.

### 2.3 MyPDB and Molecule of the Month

The RCSB PDB MyPDB service enables users to save queries, rerun queries on a regular basis, receive alerts of new query matches, tag entries of interest and add personal annotations to entries.

**Fig. 2.**
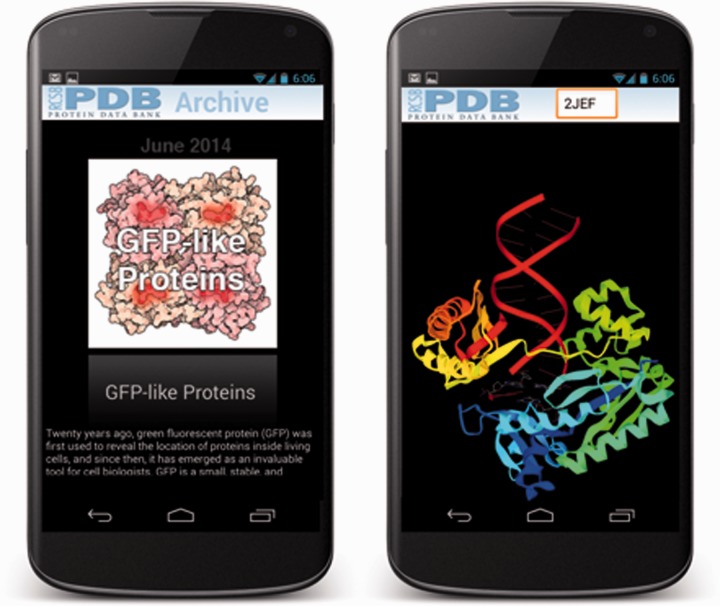
*Molecule of the Month* and molecular visualization using the embedded NDKmol on Android

The News and *Molecule of the Month* (Fig. 2) screens are Web views in the iOS and Android APIs that connect to minimal Web pages on the RCSB PDB Web server. For the news page, external links are trapped and opened either in a new modal screen (iOS) or in the default Web browser (Android). Links to example PDB structures in *Molecule of the Month* articles are trapped; clicking on the PDB ID will display that entry’s search result listing.

### 2.4 Molecular viewer

The app molecular viewer (Fig. 2) is based on an open-source molecular viewer NDKmol (http://webglmol.sourceforge.jp). NDKmol is optimized for rendering performance and efficient memory usage on mobile platforms using the graphics processing unit (GPU) and can handle large structures like ribosomes and viruses without difficulty. It supports most of the common molecular representations, such as ball-and-stick and cartoons, and coloring (by atom, by chain, by B factor, etc.).

One of the unique features of NDKmol is the fast rendering of biological assemblies and crystal packing. The viewer stores atomic coordinates and generates polygons for only one copy of the subunits. Symmetry mates are generated on the mobile device’s GPU by multiplying OpenGL’s model-view matrix with symmetry operators.

## 3 CONCLUSION

The iOS and Android clients described provide fast low-bandwidth access to data and services provided by the RCSB PDB.

*Funding*: The RCSB PDB is a member of the Worldwide Protein Data Bank and is funded by grant [NSF DBI-1338415] provided by the National Science Foundation, the National Institutes of Health, and the Department of Energy.

*Conflict of interest*: none declared.
